# HPLC-DAD-ESI/MS Identification of Light Harvesting and Light Screening Pigments in the Lake Sediments at Edmonson Point

**DOI:** 10.1155/2013/741906

**Published:** 2013-12-29

**Authors:** Rita Giovannetti, Leila Alibabaei, Marco Zannotti, Stefano Ferraro, Laura Petetta

**Affiliations:** Chemistry Section, School of Science and Technology, University of Camerino, Via S. Agostino 1, 62032 Camerino, Italy

## Abstract

The composition of sedimentary pigments in the Antarctic lake at Edmonson Point has been investigated and compared with the aim to provide a useful analytical method for pigments separation and identification, providing reference data for future assessment of possible changes in environmental conditions. Reversed phase high performance liquid chromatography (HPLC) with electrospray-mass spectrometry (ESI-MS) detection and diode array detection (DAD) has been used to identify light screening and light harvesting pigments. The results are discussed in terms of local environmental conditions.

## 1. Introduction

Depletion of stratospheric ozone since the mid-1970s has led to significant increases in ultraviolet B (UVB) irradiation over Antarctica. There is currently much interest in the survival strategies of microorganisms in hostile environments. In the remote Antarctic continent, ice-free desert regions are subject to extreme environmental stress that provides conditions for the growth of lichens, endoliths, and cyanobacteria [1, 2]. The increasingly intense flux of UVB radiation with short wavelength reaching the Antarctic terrestrial surface where cyanobacteria grow under metabolically constrained thermal and hydrological conditions has prompted the attention of geologists, biologists, biochemists, and structural chemists to draw analogies with conditions on ancient planetary surfaces [3] such as that of Mars.

During summer in the areas of Antarctica, small streams and lakes fed by glacial or snow melt water are present. This region provides many chemical data for understanding global processes such as climate change and dispersion of persistent anthropogenic pollutants because it is a symbol for a pristine environment [4]. Antarctic soils were formed in an environment described as a cold desert and characterized by extremely low temperatures which rise above 0°C for only brief periods during the short Antarctic summer, so chemical weathering processes proceed very slowly. Terra Nova Bay is part of the Ross Sea and is relatively ice-free in summer and there are more than a hundred small lakes and ponds in the area. Most of these small lakes are located along the coast and receive their sediments and water supply during the spring and summer warmer periods as melted snow because ice acts as sediment trap [5]. In the area, as in the rest of Antarctica, the soils develop and vary according to the nature of the bedrock, time, climate, and other ecological factors that control weathering.

Photosynthesis is the base of life on the earth and the development of the vast range of simple organisms existing today can often be traced back to the earliest geological times. Most life forms depend directly or indirectly on the synthetic processes which harvest the sun's energy, utilising a range of pigments such as chlorophylls and carotenoids, which not only determine the colour of each organism, but often also serve a protective role against the adverse effects of ultraviolet radiation; other photoprotective compounds are synthesized as reply to high solar irradiance.

The preservation of pigments in the sediments depends on their water solubility and physicochemical and biological characteristics, as well as on the chemical and physical qualities of the sites. In comparison to other organic material, pigments are labile compounds and the individual stability of the pigments varies. Pigments are degraded in the aquatic environment by chemical, photochemical, and biological processes. Chlorophylls contain nitrogen and are therefore more prone to being salvaged during senescence and biological breakdown than the carotenoids. Chlorophyll degradation pathways include allomerization (oxidation), demetallation (loss of the Mg), and dephytylation (loss of phytyl chain), with the five-ring phorbin being relatively stable [6]. Most of these breakdown products are detectable by regular pigment analysis. Carotenoids are found mostly in transform and are inherently more stable than chlorophylls. However, unlike chlorophylls they are often broken down to colorless compounds, by destruction of the long chain of alternating double bonds, that cannot be detected by regular pigment analysis methods [7, 8]. Knowledge of the type and variability in photosynthetic pigment composition versus lake conditions is very important for the detailed information about the history of this with reference to biotic communities [9] and of environmental factors (e.g., UV radiation or anoxia) involved in pigment transformations [10, 11].

High performance liquid chromatography (HPLC) is a reliable method for analysing algal pigments in sediments [10].

In order to better understand the environmental processes occurring in the Antarctic ecosystem, sediment samples were collected from the lake at Edmonson Point in the Victoria Land region during an Italian expedition in the period 2004–2007. In this study these sediment samples have been analyzed to determine the light harvesting and light screening pigments that are very important in Antarctic ecosystem because protect the life permitting the growth and survival of any organisms under low temperature and continuous light regime of summer.

## 2. Material and Methods

### 2.1. Site Description

The climate of central Victoria Land is a rigid Antarctic climate; the monthly mean air temperature ranges between −25.9°C (August) and −0.1°C (January). There is complete darkness between May 5th and August 10th and 24 h of light between October 10th and February 7th, while December and January show the maximum monthly mean radiation (320 and 300 W m^−2^, resp.).

*The Italian Base* is situated in Northern Foothills and is a zone of rounded off hills and covered from local little ones glacier and accumulates of snow are present near obstacles. In the outskirtses of the Base find little ones lakes or pools that in summer are opened while in the rest of the area Northern Foothills the present lakes always remain covered from the ice.

**Figure 1:**
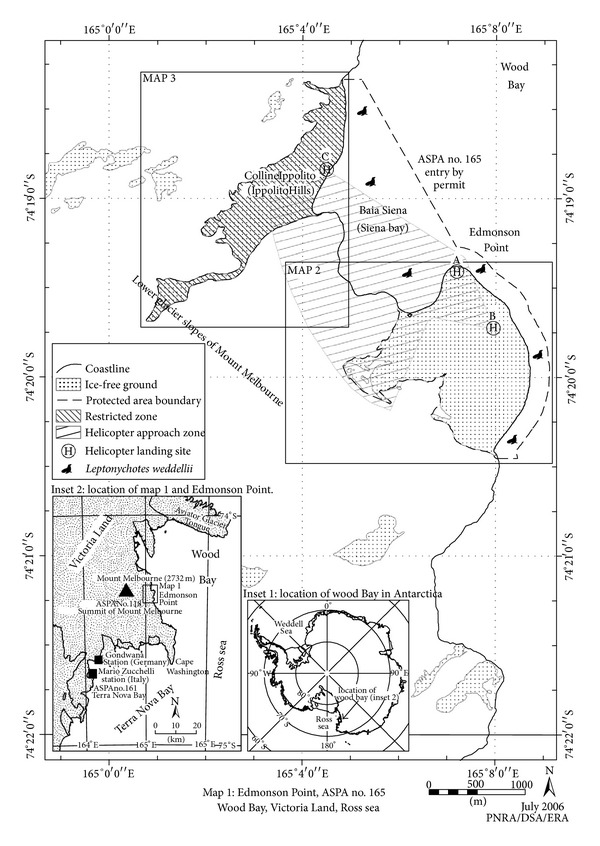
Location map of Edmonson Point in the Victoria Land.

*Edmonson Point* is located in Wood Bay, Victoria Land, Ross Sea, at the foot of the eastern slopes of Mount Melbourne, about 50 km NE of Mario Zucchelli Terra Nova Bay Station (Italy) (Figure 1).

At almost two km^2^, Edmonson Point is one of the largest of the few low-lying coastal ice-free areas in Northern Victoria Land. Edmonson Point was first identified in the 1980s as a site that could merit special protection, principally because of its rich vegetation. Italy established a station in close proximity at Terra Nova Bay in 1986-87 with increased research interest. A variety of scientific projects have been initiated, many of which are long-term with international involvement. Edmonson Point comprises hills, knolls, and moraines of volcanic materials, separated by small valleys with streams, ponds, and larger lakes. The presence of a colony of Adélie penguins, nesting skuas, and some abandoned penguin rookeries determines a patchy distribution of nutrients in terrestrial and freshwater ecosystems. The ground is generally dark in colour, which encourages rapid snow-melt in spring. In summer, most of the area is dry and the ground is covered by salt encrustations, except below late snow beds, near stream and pool margins and in flood flats. Most streams, and pools are transient. However, some long-lying snow deposits and the inland glacier margins can supply water to the larger streams and lakes from the beginning of December to the end of January. Some shallow lakes are free of ice cover for two or three months in summer, while deeper lakes are permanently frozen and are supplied by groundwater or by surface waters.

### 2.2. Sampling

Sediment samples were collected from Lake 15A at Edmondson Point during an Italian Expedition in 2004–2007. All samples were collected with plastic tools and were maintained at −24°C during all stages of storage and transport to the Italian laboratory. The samples were preserved frozen and in the dark and prior to analysis, they were freeze-dried (lyophilized) and well mixed under high vacuum (<0.1 Pa).

### 2.3. Chemicals

All solvents used were HPLC grade (Sigma-Aldrich). NH_4_Ac and MgCO_3_ were analytical reagent grade (Sigma-Aldrich). Milli-Q water was used for solution preparation.

### 2.4. Pigment Extraction

All the laboratory procedures were carried out in subdued light to minimize pigments degradation. The extraction of 20 g freeze-dried of sediment was performed in darkened flasks to prevent photo-oxidation and the breakdown of labile pigments; extraction was carried out with MeOH/acetone (10 : 90 v : v) and a small amount of magnesium carbonate in order to prevent the accidental formation of chlorophyll metabolites; this procedure was repeated until the supernatant was colourless. The extracts were filtered and concentrated using a low pressure rotary vapour at 25°C and a dry N_2_ flow. The concentrated extracts were filtered (0.45 *μ*m) before HPLC analysis. The dried extract were stored at −4°C in dark.

### 2.5. Instrumentation

Visible spectrum of total extracts was obtained with a Hewlett-Packard 8452A diode array spectrophotometer with a 1 cm quartz cell well-stoppered scanning from 200 to 800 nm.

An HPLC Agilent 1200 Series chromatograph, equipped with autoinjector and photodiode array detector (190–950 nm), was used to separate and identify individual compounds.

Separation was performed from sample aliquots of 25 *μ*L that were injected into reversed-phase columns LiChroCART (250 × 4 mm i.d. packed with LiChrospher 100, RP-18 (5 *μ*m, spherical particles)) and an ODS-hypersil (C_18_) precolumn (5 *μ*m, 20 × 4.0 mm; Hewlett-Packard).

For MS measurements the chromatograph was coupled to a 1100 MSD Hewlett Packard MSD used under ESI conditions with both positive and negative ion monitoring.

### 2.6. HPLC Separation and Analysis

The best conditions for HPLC were obtained using gradient elution with MeOH (A) and MeCN (B) as follows: 0 min, 70% A; 15 min, 80% A; 20 min, 90% A; flow rate 1 mL/min. HPLC was carried out at a controlled temperature of 28°C.

In order to obtain the best conditions for ESI-MS, the chromatograms were measured with three fragmentor voltage values (30, 70, and 150 eV) in negative and positive ion mode. All measurements demonstrated that the response was better at 70 eV in the negative ion mode and at 150 eV in the positive ion mode.

Peaks were identified by their absorption spectra at their maximum wavelength and characteristic MS fragmentations. The DAD detector was set at the wavelength of 476 or 650 nm for analysis of carotenoids and chlorophylls, respectively. Pigment quantification was performed by comparing the HPLC peak areas with those of standards (chlorophyll-*a*, chlorophyll-*b*) from DHI Water & Environment (Horsholm, Denmark) and (canthaxanthin, fucoxanthin) from the International Agency for 14C Determination VKI in Hoersholm and using published extinction coefficients [12–14] when standards were unavailable. The specific extinction coefficient used for scytonemins was 112.6 L g^−1^ cm^−1^ [15].

## 3. Results and Discussion

**Figure 2:**
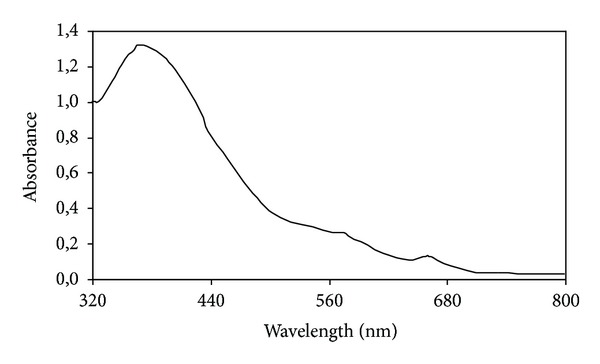
The absorbance spectra of the MeOH/acetone (10 : 90 v : v) extract of sediment lake at Edmonson Point.

The absorbance spectra of the extract of sediment is reported in Figure 2 from which the presence of chlorophyll and phaeopigments for the absorption in the range between 380 and 440 nm and at about 660 nm can be deduced. The shoulders at 450–500 nm indicate instead the presence of carotenoids.

**Figure 3:**
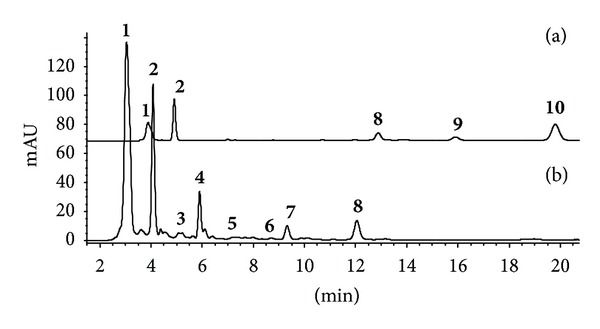
DAD chromatograms, recorded at 660 nm (a) and 476 (b), of sediment extract from Edmondson Point lake obtained using gradient elution with MeOH-MeCN (0 min 70 : 30; 15 min 80 : 20, 20 min 90 : 10); peak numbers correspond to pigments listed in Table 1.

The results of HPLC analysis of sediments of Lake 15A at Edmonson Point are reported in Figure 3. The chromatogram obtained at 650 nm (Figure 3(a)) showed the presence of five pigments, while in that obtained at 476 nm (Figure 3(b)), eight pigments were observed. ESI-MS analysis of the mixture combined with on-line DAD electronic spectra allowed identification of the major components and their percentage composition (Table 1).

**Figure 4:**
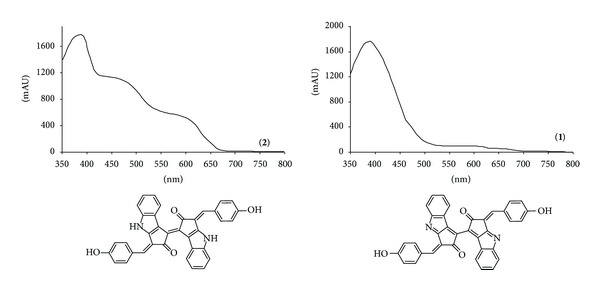
UV-vis spectra and formulae of scytonemin (**2**) and its reduced form (**1**).

The two components eluting at 4.7 and 3.1 min were assigned as scytonemin and its reduced form [10] (**2, 1**) from [M+H]^+^ at *m*/*z* 545.5 and 547.7, [M+Na]^+^ at *m*/*z* 567.5 and 569.5 and [M−H]^−^ at *m*/*z* 543.5 and 545.5, respectively, and from the UV-vis spectra (Figure 4) that show the absorption over relatively wide range of wavelength from 325 to 425 nm.

**Figure 5:**
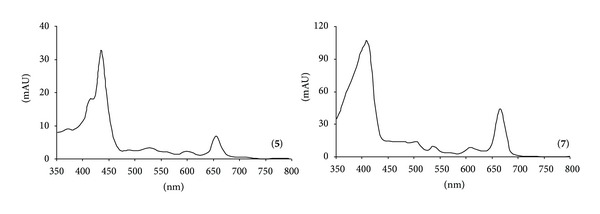
UV-vis spectra of phaeophytin *a* (**5**) and phaeophytin *b* (**7**).

Peaks **5** and **7** were attributed to pheophytin *b* and *a* from [M−H]^−^ at *m*/*z* 884 and 870, respectively, and from the relative UV-vis absorption bands (Figure 5).

**Figure 6:**
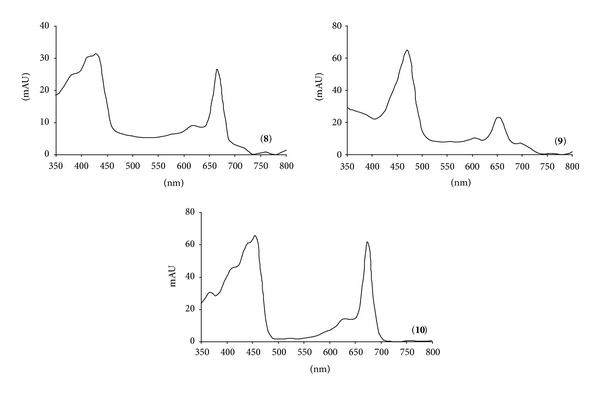
UV-vis spectra of chlorophyll *b* (**8**), bacteriochlorophylls *b* (**9**), and chlorophyll *a* (**10**).

Peaks **8**, **9**, and **10** showed [M−H]^−^ acetone adducts at *m*/*z* 965, 870, and 951; these ions, together with spectral data (Figure 6) and literature information, allowed us to recognize the parent pigments chlorophyll *b*, bacteriochlorophylls *b*, and chlorophyll *a*, respectively.

**Figure 7:**
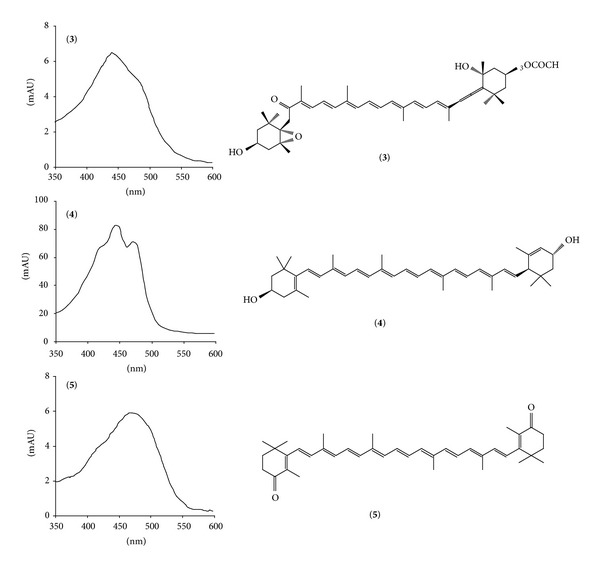
UV-vis spectra of fucoxanthin (**3**), lutein (**4**), and canthaxanthin (**5**).

The other peaks (**3**, **4**, and **6**) showed typical carotenoid UV-vis spectra (Figure 7); [M−H]^−^ at *m*/*z* 657 and [M+Na]^+^ at *m*/*z* 681 for peak **3** were attributable to fucoxanthin, while [M−H]^−^ at *m*/*z* 567 and [M+H]^+^ at *m*/*z* 569 were attributable to lutein for peak **4**. The [M−H]^−^ ion at *m*/*z* 563, together with [M+H]^+^ and [M+Na]^+^ ions at *m*/*z* 565 and 587,was assigned to canthaxanthin (peak **6**).

Scytonemin is an ultraviolet screening, photostable sheath pigment produced under conditions of high solar irradiance by cyanobacteria (blue-green algae) and accumulates in extra-cellular sheaths for photoprotection against DNA damage [16, 17]. Scytonemin allows the renowned survival ability of these bacteria [18] and is produced in response to elevated levels of UV-A radiation; its production is governed by a complex mechanism in which a number of environmental factors are implicated. The UV/Vis absorption profile of scytonemin is such that it absorbs UV-A radiation strongly while transmitting the wavelengths of light that are absorbed by the photosynthetic apparatus of cyanophyta.

In addition, the photobleaching of chlorophyll *a* by UVA radiation was retarded in scytonemin-containing cyanobacterial sheaths. It was noted also that there seemed to be a direct relationship between the production of the scytonemin in cyanobacteria and the exposure to UV flux. The scytonemin molecule is not photodegraded and is believed to have a long-term persistence in terrestrial crusts or dried cyanobacterial cultures [16, 17, 19].

The reduced form of scytonemin called Red Scytonemin is another UV-absorbing compound that is stable over long time scales and is formed by transformation of scytonemin under anoxic reducing conditions after loss of cell integrity of cyanobacteria [16]. Also canthaxanthin [20] is UV-inducible pigments that protects organisms against the deleterious effects of UV radiation [16].

Because scytonemin, its reduced form and the carotenoid canthaxanthin were detected in the sediment of Edmonson Point, it is possible to assume the presence of colonial cyanobacteria in the water column at this site.

Fucoxanthin is an indicator pigment for diatoms, chrysophytes, and prymnesiophytes [21], while chlorophyll *a* is indicative of the presence of autotrophic benthic algae.

The presence of chlorophyll *b* and lutein can be attributed to an input from green algae (chlorophyta) and perhaps to the vegetation present in the area. Lutein is a lipophilic molecule, is the most abundant carotenoid in photosynthetic plant tissues that has distinctive light harvesting properties, and is generally insoluble in water, so may be present in the sediment as an aggregate. Because the polyenic chain is susceptible to oxidative degradation via light or heat and is unstable in acid, its presence is indicative of anaerobic and not acidic conditions during its deposition in the sediment.

**Figure 8:**
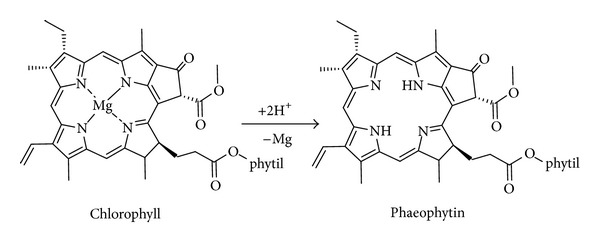
Reaction that occurs in the chlorophyll demetallation for the formation of phaeophytin.

Phaeophytin-*a* and *b* are metal-free chlorophylls, acidic conditions promoting displacement of the metals (Figure 8), so their presence is indicative of pH change at the time of their deposition.

Bacteriochlorophyll-*c* is found only in green photosynthetic bacteria, organisms that contain a special antenna complex known as a chlorosome [22]. It has a natural tendency to form large aggregates with highly organized helical structures that absorb light very efficiently, with fast energy transfer to some membrane bound energy acceptors; it is highly probable that it was deposited in such an aggregate form.

The quantitative analysis of pigments (Table 1) revealed that the major components are in the order, lutein, scytonemins, fucoxanthin, and canthaxanthin, respectively, and only small amounts of chlorophyll and its degradation products are present.

Scytonemins is about 10 times cyanobacterial carotenoids (canthaxanthin) confirming high biological receipt of UV radiation while cyanobacterial carotenoids are about 6 times total chlorophyll-*a* showing high cyanobacterial receipt of photosynthetically active radiation. Comparison between scytonemins with total chlorophyll that is about 60 confirms high proportion on photoprotection versus photosynthetic production.

## 4. Conclusions

Detailed studies of chlorophylls, carotenoids, and their derivatives in aquatic sediments and their isolation and identification may permit to use fossil pigments to reconstruct past changes in lake production. Thus, the type of fossil pigments can reflect the sedimentation history and simultaneously give indirect information about the dynamics of the abundance of algae in lakes.

Because of the complexity of the pigments mixtures which typically occur in sediments, the separation of the individual components can be problematic; in this paper we have shown that the combination of HPLC/DAD/ESIMS in negative and positive ionization modes allowed the separation and the conclusive identification of sedimentary pigments extracts from lake 15A at Edmonson Point. This method allowed us to determine the characteristics of several photosynthetic pigments that are present in the lake sediments because the cold climate in the area favours their preservation.

The pigments could be classified into four families of compounds: light screening pigments as scytonemins and light harvesting pigments as carotenoids and chlorophylls other than phaeophytins (its degradation pigments). Estimates of these pigments suggest a stronger UV stress in the Lake 15A at Edmonson Point.

This chemical approach may be useful in the extensive studies of these sites in the temporal and spatial variations in composition of sedimentary pigments as responses to changing environmental conditions.

## Figures and Tables

**Table 1:** Pigments in Edmonson Point lake sediments identified using DAD and ESI mass spectra.

Peak	Time (min)	Pigment	Main UV-vis bands (nm)	ESI+ (*m*/*z*)	ESI− (*m*/*z*)	Composition %
**1**	3.1	Reduced scytonemin	385–450–590	[M+H]^+^ 547.5 [M+Na]^+^ 569.5	[M−H]^−^ 545.5	11.90
**2**	4.7	Scytonemin	385	[M+H]^+^ 545.5 [M+Na]^+^ 567.5	[M−H]^−^ 543.5	8.67
**3**	5.1	Fucoxanthin	440–470	[M+H]^+^ 681	[M−H]^−^ 657	9.92
**4**	5.9	Lutein	420–450–480	[M+H]^+^ 591	[M−H]^−^ 567	64.59
**5**	6.1	Pheophytin b	(420) 450–475–665	—	[M−H]^−^ 884	0.30
**6**	7.8	Canthaxanthin	478	[M+H]^+^ 565 [M+Na]^+^ 587	[M−H]^−^ 563	2.07
**7**	9.3	Pheophytin a	475–665	—	[M−H]^−^ 870	0.70
**8**	12.1	Chlorophyll b	465–600–650	—	[M−H]^−^ 965^a^	0.65
**9**	15.1	Bacteriochlorophyll-c	370–420–610–660	—	[M−H]^−^ 870^a^	0.24
**10**	19.2	Chlorophyll a	370–415–435–610–660	—	[M−H]^−^ 951^a^	0.30

^a^Acetone adduct.
